# Health Service Activity Standards and Standard Workloads for Primary Healthcare in Ghana: A Cross-Sectional Survey of Health Professionals

**DOI:** 10.3390/healthcare9030332

**Published:** 2021-03-16

**Authors:** James Avoka Asamani, Christmal Dela Christmals, Gerda Marie Reitsma

**Affiliations:** 1 Centre for Health Professions Education, Faculty of Health Sciences, North-West University, Potchefstroom Campus, Building PC-G16, Office 101,11 Hoffman St., Potchefstroom 2520, South Africa; asamanij@who.int (J.A.A.); gerda.reitsma@nwu.ac.za (G.M.R.); 2 Intercountry Support Team for Eastern and Southern Africa, Health Workforce Unit, Regional Office for Africa, World Health Organisation, 82-86 Cnr Enterprise/Glenara Roads, Harare CY 348, Zimbabwe

**Keywords:** health service standards, standard workload, activity standards, primary health care, health workforce planning, human resources for health, workload indicators of staffing need (WISN)

## Abstract

The attainment of health system goals is largely hinged on the health workforce availability and performance; hence, health workforce planning is central to the health policy agenda. This study sought to estimate health service activity standards and standard workloads at the primary health care level in Ghana and explore any differences across health facility types. A nationally representative cross-sectional survey was conducted among 503 health professionals across eight health professions who provided estimates of health service activity standards in Ghana’s Primary Health Care (PHC) settings. Outpatient consultation time was 16 min, translating into an annual standard workload of 6030 consultations per year for General Practitioners. Routine nursing care activities take an average of 40 min (95% CI: 38–42 min) for low acuity patients; and 135 min (95% CI: 127–144 min) for high dependency patients per inpatient day. Availability of tools/equipment correlated with reduced time on clinical procedure. Physician Assistants in health centres spend more time with patients than in district hospitals. Midwives spend 78 min more during vaginal delivery in health centres/polyclinics than in district/primary hospital settings. We identified 18.9% (12 out of 67) of health service activities performed across eight health professional groups to differ between health centres/polyclinics and district/primary hospitals settings. The workload in the health facilities was rated 78.2%, but as the workload increased, and without a commensurate increase in staffing, health professionals reduced the time spent on individual patient care, which could have consequences for the quality of care and patient safety. Availability of tools and equipment at PHC was rated 56.6%, which suggests the need to retool these health facilities. The estimated standard workloads lay a foundation for evidence-based planning for the optimal number of health professionals needed in Ghana’s PHC system and the consequent adjustments necessary in both health professions education and the budgetary allocation for their employment. Finally, given similarity in results with Workload Indicators of Staffing Need (WISN) methodology used in Ghana, this study demonstrates that cross-sectional surveys can estimate health service activity standards that is suitable for health workforce planning just as the consensus-based estimates advocated in WISN.

## 1. Introduction

The extent of health service coverage and its quality anywhere is primarily hinged on the health workforce’s availability and quality [1,2]. Therefore, as countries continue to push for Universal Health Coverage (UHC) and the Sustainable Development Goals (SDGs), planning for the health workforce has become a central part of the health policy agenda. One component of the health workforce planning process involves determining the optimal number and mix of health workers required to serve a given population. Three broad approaches, population ratios, service demand/utilisation-based, and population needs-based approaches, have been used in planning the health workforce. Underlying each of these approaches is an explicit (as in needs-based and utilisation-based approaches) or implicit (as in population ratios) assumption about some measures of health worker productivity [3,4]. The Workload Indicators of Staffing Needs (WISN) tool, which is part of the service demand/utilisation-based approaches, is developed by the World Health Organisation (WHO) and widely used [5,6,7]. In the WISN manual, WHO defined standard workload as the amount of work of a particular service delivery activity that one health professional who is well trained and reasonably motivated could perform in a year if the health professional dedicated all his/her working time to delivering that service [5,6,7]. This depends on how much time a health professional has available in a year, and the professional standard time required to deliver one unit of his/her health service tasks (see Equations (2) and (3)). In the WISN methodology, staffing requirements to cover a health service activity is derived by dividing the observed workload(or count of service utilisation) for that activity by its standard workload [8,9,10]. Similarly, in the population needs-based methodology for health workforce requirements, estimates of anticipated workload (based on population size, demographics, health status indicators and planned or otherwise required type of services) are divided by the health worker productivity standard to derive the staffing requirement. Thus, both the service demand/utilisation-based approach and the needs-based approach have similarity in the denominator; they use some measure of productivity or standard workload but only differ in whether anticipated workload (needs) or observed workload levels (service utilisation) should be used as the numerator. Additionally, there is an emerging approach in predicting health professionals’ workload using health expenditure and budgets [11]. With the standard workloads for health professionals established, it would also be feasible to estimate the required health professionals using the predicted workloads from this emerging approach. 

Proponents of the needs-based approach have not had consensus on the best proxy measure of health worker productivity; hence different measures have been used in previous estimates, mostly with validity concerns [12,13,14]. Asamani et al. [15] have argued that the concept of standard workload used in the WISN methodology appears to be a reasonably valid measure of a productivity standard that could be applied in a needs-based framework for health workforce planning. This will also allow for a concurrent application and comparability of results between the two approaches since the only divergent variable will be the measure of workload (i.e., observed workload versus predicted/anticipated workload). 

Although all WISN studies have inherently elicited service (activity) standards and determined the standard workloads, most papers have focused on reporting the staffing estimates and gaps, which tend to be the WISN study outcome of policy interest. Thus, it has become necessary to systematically elicit and document the service standards and standard workloads of various health workers, which becomes an important reference standard for health workforce planning and management.

Ghana operates a multi-tier gatekeeper health system where community-based health planning and service (CHPS) compounds/zones serve as the first contact of the health system. CHPS are mainly manned by nurses who live within the communities to serve a population of 5000 or 750 households in sparsely populated areas [16]. At this level, the main services provided are preventive, treatment of minor illnesses and maternal care, including deliveries where there is a midwife. The next level of the health system is health centers that serve as the first referral level health facility at the sub-district level for populations up to 200,000 but can be enhanced in urban areas to serve larger populations, in which case they are designated as polyclinics [17]. At these levels, a wide range of services are provided on an outpatient basis and with facilities for short-term admission for observation (not more than 24–48 h). Most health centres are headed by physician assistants, while polyclinics usually have a medical officer (general practitioner). District (primary) hospitals are established in administrative district capitals or for populations between 100,000 and 200,000, where they provide a range of outpatient and inpatient services. Administrative regions have regional hospitals that are equipped with specialist facilities for the secondary level of care. However, in large regions and metropolis, secondary hospitals may be designated for populations of at least 1.2 million. At the health system’s apex are tertiary hospitals (usually teaching hospitals affiliated with universities) that provide super-specialised health services and have a mandate for teaching and cutting-edge research [17]. 

Over the years, Ghana’s health workforce situation dramatically improved from 1.07 doctors, nurses, and midwives per 1000 population in 2005 to 2.56 per 1000 population in 2018 [18], and with a capacity to produce more than 25,000 health professionals of various categories per year. However, various reports have shown that Ghana’s health workforce situation is still sub-optimal and plagued with inequitable geographical distribution, sub-optimal productivity, and inefficiencies [19,20]. Determining the optimal need for health professionals and using the same to plan health service delivery, the production, recruitment, and distribution of health professionals has been a delicate issue with vested interests recently [21,22]. 

Ghana completed a WISN study in 2018 in which the activity standards were elicited using an open-ended questionnaire in which conveniently selected health professionals across health facilities provided information on their main activities and the corresponding time for accomplishing each of them [23]. Aimed to achieve a technical consensus, an initial group of health professionals provided a list of health service activities they perform, and the corresponding time spent on each patient. These were then collected and sent to the next health facility, where the completed tool was given to another batch of health professionals (in the same category) to indicate if they agreed with the previous batch of health professionals’ proposal. The process continued until a near consensus was achieved where no new issues were raised by the subsequent batches of health professionals [24]. Although some of the established standards were cross-validated with time-motion observations, the approach arguably inhibited divergent views in the setting of standards. With the consensus-based approach adopted then, it was not possible to measure the level of uncertainty or practice variations that may be inherent across rural and urban areas, and the different types of health facilities. To address this concern, we undertook a cross-sectional study to elicit the activity standards of eight categories of health professionals at primary health care settings in Ghana. This study was, therefore, aimed at (a) systematically estimating service standards (the mean estimates of time spent on health service activities) alongside the level of uncertainties or practice variation (at 95% confidence interval); (b) the resulting standard workload per year and examined any differences across health facility types and other characteristics; and (c) explore the relationship between health service activity standards, and workload levels and availability of tools/equipment. 

## 2. Methods

### 2.1. Study Design

A cross-sectional survey design was adopted for this study to obtain information on the prevailing practice of health professionals under the given circumstances of their health facilities. 

### 2.2. Population

The target population in this study included health professionals (general practitioners, nurses, midwives, physician assistants, biomedical scientists, pharmacists, pharmacy technicians and nutritionists/dieticians) working in Primary Health Care (PHC) settings (primary hospitals, health centres and CHPS compounds/zones) in Ghana. 

The inclusion criteria were full-time health professionals of the GHS with the requisite practising license from the relevant professional regulatory council who were working in either a primary/district hospital, or a health centre/polyclinic or a CHPS zone/compound and with a minimum of one-year post-qualification working experience in their duty post at the time of the study. Professionals with less than one year post-qualification working experience were assumed to be less proficient in the performance of key tasks [25] and could bias the standard time estimation if they were included. Health professionals who were on internship or relief duty (not their permanent post) were excluded, same for health professionals who were performing managerial or other duties different from their core professional training for more than 50% of their time. 

### 2.3. Sample Size and Sampling Technique

#### 2.3.1. Sample Size

Available data from the GHS [26] showed that in April 2020, the three (3) sampled regions had about 58,984 of the health professionals of interest in this study (general practitioners, nurses, midwives, physician assistants, biomedical scientists, pharmacists, and nutritionists/dieticians) working at the PHC level. Using this total number as the accessible population and an alpha level of 0.05, the minimum sample size was estimated to be 397 using the simplified Yamane’s sample size formula (Box 1) [27]. To account for anticipated non-response, an assumed rate of 15% was added based on previous experiences with surveys involving health professionals in Ghana [28]. The adjusted sample size was a minimum of 456 health professionals to have sufficient representation of each health professional category to allow for inference [29]. An all-inclusive sample was used for the health professional categories with a total staff of thirty (30) or less in any of the three regions. Thus, the overall nationally representative sample estimated to be at least 591 health professionals across the three (3) regions.

Box 1 **Sample size determination. Source: Yamane (1967) [27].**

(1)$$n=\frac{N}{1+N{\left(e\right)}^{2}}$$

where: *n* = required sample size
*N* = Accessible population
*e* = alpha level or significance level
Thus,
$$n=\frac{58984}{1+58984{\left(0.05\right)}^{2}}\;\approx \;397$$
Adjusted sample size = 612

#### 2.3.2. Sampling Technique

A multistage stratified sampling approach was used to recruit a nationally representative sample [30]. First, the sixteen (16) regions of Ghana were geographically divided into three (3) main clusters: southern, middle, and northern zones. One region from each of these clusters was randomly selected through balloting in which the Greater Accra region in the southern zone, Bono East region in the middle zone, and Upper East region in the northern zone were selected for the study. 

Each geographical stratum (represented by the regions) was allocated a sample proportional to its share of the national stock of the respective occupational groups of health professionals of interest (see Table 1). Similarly, in each stratum (region), the allocated sample was further proportionally allocated to the health facility types (primary/district hospital, health centre and CHPS zone). The health facilities were then randomly selected using Microsoft^®^ Excel random numbers. In each health facility, the proportionally allocated sample size was further divided proportionally for the various health professional categories of interest in that health facility. For the health professionals, a simple random sampling using Microsoft^®^ Excel was used to select those to contact from the facility’s staff list (nominal roll). Whenever a health professional who has been randomly selected was contacted and he/she declined to participate in the study, others of similar health professional category in the facility (if available) were randomly sampled as a replacement until the required sample from that facility was exhausted. 

### 2.4. Recruitment of Participants

Following ethical approval from the ethics review committees, permission was sought from the Director-General of GHS, which was granted, and an introductory letter was written from the office of the Director-General to the respective Regional Directors of Health Services (RDHS) to allow the study to proceed and serve as regional gatekeepers. The RDHS, in turn, informed the selected health facilities of the study to grant access to the participants. 

In each hospital, the facility quality assurance or research coordinator served as mediators to facilitate the identification and access to potential participants. A research information sheet was handed out to prospective participants individually at their various units or wards by the research coordinator or the facility’s quality assurance coordinator. However, in health centres and CHPS compounds, the staff are usually very few and with no quality assurance or research coordinators. In these settings, the researchers approached the team leader in the facility to identify one of the staff who was not within the study’s inclusion criteria and had no power relationship with the other staff to serve as a mediator. The mediator supported the researchers to contact the rest of the staff to hand out the information sheet. Health professionals who met the inclusion criteria and voluntarily agreed to participate in the study were given a voluntary consent form to carefully study, ask the necessary questions and sign if they willing to take part in the study. 

Upon signing informed consent, willing participants were given the questionnaire and guided as needed to complete it via Data Analytics^®^, an online data collection platform with end-to-end encryption widely used for survey data collection. The geographical coordinates of the location where the participant completed the data collection tool were automatically recorded and linked to the data to enable audit. The data collection was conducted from 21 September 2020 to 21 December 2020.

### 2.5. Data Collection Tool

The data collection tool was adapted from the job components tool used for data collection in the Workload Indicators of Staffing Needs (WISN) study in Ghana [31,32]. The tool collected background information on the health facilities in which the health professionals work and that of the health professionals themselves, and the average time it takes health professionals to perform their main health service delivery activities. As the data collection tool was intended to measure the amount of time health workers spend in undertaking different health service delivery tasks, the data collection tool was designed to reflect the job description of the various health professionals and was scrutinised by peers and subject matter experts (SME) in the health professions whose feedback was used to make adjustments to address the objectives of the study better and ensure content validity. 

### 2.6. Ethical Considerations

The study was reviewed and approved by the North-West University’s Health Research Ethics Committee (NWU-00416-20-A1) and the GHS Ethics Review Committee (GHS-ER17/07/20) before the commencement of the study. Following ethical approval from both institutions, permission and introductory letters were obtained from the Director-General of GHS to the Regional Directors of Health Services (RDHS) and heads of health facilities to access participants. Participation was entirely voluntary, and the informed consent was obtained by an independent person who had no power relation with the participants. No individual information of health professionals or the facility they work in have been reported; instead, aggregate data is analysed and reported.

### 2.7. Data Processing and Analysis

The raw data was downloaded from the online Survey Analytics^®^ Software to Microsoft Excel^®^ for cleaning, after which it was imported into SPSS^®^ version 26 for analysis. Descriptive analysis with estimates of uncertainty was undertaken mainly to estimate the average time taken by various health professionals to perform their service delivery activities and the 95% confidence interval around the point estimates. Inferential analysis in the form of an independent *t*-test [29,33] was conducted to examine if there were significant differences between district/primary hospitals’ activity standards and those of the health centres/polyclinics. Pearson’s correlation analysis [29,33] was also conducted to examine any association between self-reported workload levels and the estimated activity standards. 

### 2.8. Computing Standard Workload from Service Standards and Available Working Time

As defined in the WISN manual [7], the standard workload is a function of two components: (a) the Service Standard (SS) for the activity to be performed, which is defined as the average time that it will take well-trained and motivated health professional to complete the health service activity to acceptable professional standards within the context of the jurisdiction and (b) the Available Working Time (*AWT*)—the time a health worker has available in one year to do his/her work, taking into account all absences (Equation (2)).

AWT = A − (B + C + D + E + F) (2)

where:

*AWT* is the total available working time
*A* is the number of days in a year = 365
*B* is the number of days off for public holidays in a year
*C* is the number of days off for annual leave in a year
*D* is the number of days off due to sick leave in a year
*E* is the number of days off due to other leave, such as training, etc., in a year.
*F* is the number of weekend days or off-duty days

(3) $$SW_{n,y}=\frac{AWT_{n}}{SS_{y,n}}$$

where:

*SW**_n,y_*** is the standard workload for health professional category *n* when performing health service activity *y*.
*AWT**_n_*** is the available working time of the health professional category *n.*
*SS**_y,n_*** is the Service Standard or the time it takes a well-trained health professional of type *n* to deliver the service activity, *y*.

## 3. Findings

### 3.1. Demographic Characteristics of the Participants and Health Facilities in Which They Work

A total of 503 valid questionnaires were received from participants out of the estimated sample size of 612, representing a response rate of 82.2%. The participants’ ages ranged from 20–60 years, with an average of 33 years (±7.02 years). The majority of the health professionals (*n* = 267, 53%) were between the ages of 30–39 years, followed by those in the 20–29 years age group (*n* = 166, 33%). About 86% of the health professionals were below 40 years which signifies the youthful nature of the health professionals. Only 4.2% (*n* = 21) were 50 years or older (Table 2).

Most participants were clinical nurses—Registered General Nurses and Enrolled Nurses (*n* = 219, 43.5%) followed by Community Health Nurses (*n* = 65, 12.9%) and Laboratory Scientist/Technicians (*n* = 56, 11.1%) while Nutritionist/Dietician were the least (*n* = 6, 1.2%). Almost one (1) in three (3) of the health professionals (*n* = 158, 31.4%) had post-secondary school certificate qualification in their respective professions, a similar proportion (*n* = 158, 31.4%) had either a university degree or post-graduate qualification. About 26% (*n* = 133) had diploma qualifications and nearly 5% (*n* = 24) indicated having other qualifications which included fellowship with medical colleges, higher national diplomas, and doctorate degrees. 

The health professionals had diverse working experience ranging from one (1) to forty-four (44) years, with the average being about 7 years (mean = 6.8 years, SD = 3.41 years). Additionally, at the time of the study, the health professionals, on average, had been working in their health facilities for nearly 4 years (mean = 3.6 years, 95% CI: 3.4–4.0 years (Table 3).

Consistent with the distribution of the health workers in the country, most of the participants were from the Greater Accra region (*n* = 235, 46.7%), followed by the Upper East region (*n* = 170, 33.8%) and the rest from Bono East region (*n* = 98, 19.5%). Additionally, more than half were based in primary/district hospitals (which are usually in the urban parts of the districts), whereas the health centres/polyclinics and CHPS had 37% (*n* = 186) and 10.1% (*n* = 51), respectively. The vast majority (61.2%, *n* = 308) considered the location of their health facilities to be urban areas, while 38.7% thought they were either working in a rural (12.3%) or semi-urban (26.4%) area.

### 3.2. Estimated Health Service Activity Standards 

We summarised the average time spent by the different health professionals on their respective health service activities and reported the level of uncertainty in the estimated time using standard deviation, standard errors, and the 95% confidence interval (CI) around the mean. The activity standards are presented in more detail in Table 4. 

#### 3.2.1. General Practitioners (Generalist Doctors)

Across the primary health care (PHC) settings, General Practitioners spent an average of 16 min (95% CI: 13–19 min) on the assessment, diagnosis and treatment of new patients in the outpatient clinic but 9 min (95% CI: 8–11 min) for outpatients on follow-up visits. Reviewing patients who are on admission (i.e., daily ward rounds) took an average of 15 min (95% CI: 12–18 min), while minor surgeries, including repair of lacerations, incisions and drainage, etc., took 19 min (95% CI: 16–23 min). Major surgeries within the PHC context, including caesarean section, laparotomy, herniorrhaphy, etc., took an average of 64 min. However, given wide variations in these procedures’ complexity, there was also considerable uncertainty in the estimated time spent. Therefore, when the 95% confidence interval is considered, major surgeries at the PHC level could be done within 44 min or take as long as 84 min. 

#### 3.2.2. Physician Assistants (Medical) 

The direct patient care activities performed by Physician Assistants (PAs) at the PHC settings closely mirrored those of the General Practitioners, except major surgeries, which they are, by law and job description, not mandated to undertake. PAs spent an average of 16 min (95% CI: 13–18 min) on the consultation of new outpatient but an average of 10 min (95% CI: 8–11 min) for follow-up/review of old outpatient cases. However, unlike the General Practitioners who spend about 19 min on minor surgical procedures, the PAs spent about 25 min (95% CI: 22–28 min). When the 95% confidence intervals are taken into consideration, there is no significant difference between them (as the confidence intervals overlap).

#### 3.2.3. Midwives 

For midwives, antenatal care consultation took an average of 22 min (95% CI: 18–26 min). For postnatal consultations, an average time of 19 min was spent on each patient by midwives (95% CI: 17–27 min); 16 min (95% CI: 12–21 min) for non-invasive family planning services; and 39 min (95% CI: 33–45 min) for invasive family planning procedures. Additionally, for each supervised vaginal delivery, midwives spend an average of 131 min which could be as short as 101 min or as long as 160 min, when the uncertainty in the estimate (95% confidence interval) is taken into account. For each day that a patient stays on admission after delivery, midwives spend about 30 min on each of the mother and the newborn for routine care but as much as 45 min if there is a pregnancy complication. Besides, patients received roughly 23 min (95% CI: 21–25 min) of patient education and counselling from midwives. In addition to the direct patient care activities, midwives spent about 31 min (95% CI: 25–36 min) per day on handing over (and taking over) from one shift to another—this represents 6.5% of the daily working time of midwives.

#### 3.2.4. Clinical Nurse (Registered General Nurse and Enrolled Nurse) 

The survey revealed that clinical nurses spend up to 10 min on history taking and checking vital signs in out-patient clinics, but where they were conducting a full out-patient consultation, an average of 13 min (95% CI: 12–14 min) is spent on each outpatient. Patients requiring admission into inpatient wards consumed about 19 min (95% CI: 18–20 min) of nurses’ time for the admission process while the process of discharging them also took about 16 min (95% CI: 14–17 min), in addition to an average of 18 min nurses spend on patient education and counselling. Preparing a patient for surgical procedures (which is considered not part of routine inpatient care) took an average of 26 min (95% CI: 23–28 min) of nurses’ time, whereas the post-operative management of surgical patients (excluding routine nursing care) was estimated to be an average of 42 min (95% CI: 38–46 min) per patient per inpatient day.

Depending on the patient’s acuity level, there is a substantial variation in the time nurses spend in routine nursing care activities, including monitoring vital signs, administering prescribed medications, and supporting patients in their daily living activities. Low dependent patients received an average of 40 min per inpatient day (95% CI: 38–42 min), while patients with moderate dependency required some 43 min (95% CI: 40–46 min) per inpatient day. Expectedly, high dependency patients required an average of 135 min per inpatient day at the primary care level, but the true estimate could be between 127 min and 144 min when the 95% confidence interval is taken into consideration. In addition to the various direct patient care activities, clinical nurses, on average, spend some 25 min each day on handing/taking over and another 32 min on writing daily reports. Thus, nearly 12% of nurses’ daily working time could be spent on handing/taking over and report writing (not including the recording of nursing actions that are part of each nursing procedure).

#### 3.2.5. Preventive Nurse (Community Health Nurse) 

The average time to provide family planning services by Community Health Nurses (CHNs) at the PHC level was about 14 min (95% CI: 12–17 min). In line with national policy, CHNs at the CHPS compounds undertakes consultation and treatment of minor ailments, which takes about 12 min (95% CI: 10–14 min) per patient. A similar amount of time (an average of 12 min, 95% CI: 10–14 min) is spent per each school child anytime school health activities are conducted. Both immunisation and growth monitoring each took an average of 6 min, whilst home visits took about 23 min (95% CI: 20–26 min) per household visited (excluding travel time for the home visiting). Additionally, CHNs spend 17 min per day to check and report on the maintenance of the cold chain for their vaccines.

#### 3.2.6. Nutritionists and Dieticians

With a limited sample, it is estimated that Nutritionists and Dieticians spend about 13 min per patient on nutritional status assessment, and patients requiring nutritional interventions receive about 27 min (95% CI: 12–42 min) per visit or inpatient day while an additional 22 min is spent on each patient for education and counselling. It is worth emphasising that due to the limited sub-sample of only 6 nutritionist/dieticians in the study, these estimates must be interpreted with caution.

#### 3.2.7. Laboratory Scientist/Technicians 

On average, obtaining and processing specimens (samples) for laboratory examination took 9 min (95% CI: 8–11 min) per patient. On actual tests, undertaking a full blood count using an automated analyser machine took about 6 min (95% CI: 5–7 min). Performing a rapid diagnostic test for malaria, HIV, and similar ones took an average of 17 min (95% CI: 16–18 min), the same for performing a batch of blood chemistry test using automated machines. Culture and sensitivity analysis, a procedure seldomly performed at the PHC level, was estimated to take about 74 min but spread over a period of 2–4 days. When the uncertainty in the estimate is taken into account, culture and sensitivity analysis could take only 67 min or as much as 81 min. Besides the direct patient care activities performed on patients/sample, laboratory scientist/technicians spend roughly 10–13 min per day writing some summary reports and up to 8 h per month (upper confidence limit) on blood donation campaigns.

#### 3.2.8. Pharmacists/Pharmacy Technicians 

The time spent by Pharmacists/Pharmacy Technicians in auditing and dispensing prescribed medicines for outpatients (mean = 7, minutes 95% CI: 6–9 min) and inpatients (mean = 7 min, 95% CI: 6–8 min) were similar; same for the refilling of medications for patients with chronic illnesses. However, patient education and medication adherence counselling took 13 min (95% CI: 11–15 min). In addition to the direct individual patient care activities, Pharmacist/Pharmacy Technicians spend roughly 14 h per week or 35% of their working time reconstituting powdered preparations (3 h per week), managing pharmaceutical stock (7 h per week), and attending clinical meetings (4 h per week).

### 3.3. Estimated Standard Workload per Year for the Health Service Activities 

Using Equation (2), we computed the available working time (AWT) for each health professional (Supplementary Materials), which show that General Practitioners and PAs have 198 working days in a year (equivalent to 95,040 min) while the other categories have approximately 200 available working days per year which is equivalent 96,000 min per year. Using the AWT in minutes as the numerator, we derived the standard workloads for each of the activity standards (see Equation (3)), which are crucial inputs for not only workforce planning but also productivity and efficiency management and analysis.

As shown in Table 5, if a General Practitioner is dedicated to only outpatient consultations, he/she could be reasonably expected to attend to 6030 new patients per year (or 10,444 follow-up cases per year). Given a marginal difference in the activity standard, PAs could see about 6108 new outpatients per year. The average number of deliveries that could be undertaken by a midwife in the PHC context of Ghana is estimated to be about 735, but the true value may lie somewhere between 598 and 951 per year. For patients with low-to-moderate dependency (as usually seen in PHC settings), a Clinical Nurse can be expected to nurse between 2078 and 2549 per year, which translates into a nurse-patient ratio of one nurse to 5–7 inpatients. A well-trained CHN could also provide roughly 16,000 (between 13,793 and 18,714) routine immunisations per year. A Laboratory Scientist/Technician could also perform some 5511 (or between 5199 and 5863) rapid diagnostic tests for Malaria, HIV or similar ones per year. Finally, a Pharmacist/Pharmacy Technician can audit and dispense 13,205 (or between 11,096 and 16,304) prescriptions in a year, while a Nutritionist/Dietician could undertake 7202 nutritional status assessments per year. Table 5 provides the details on the mean standard workloads with confidence intervals for all the health service activities included in the study.

### 3.4. Differences in Activity Standards between Health Centres/Polyclinics and District/Primary Hospitals

The services rendered at CHPS compounds are usually basic and performed by CHNs and some clinical nurses. However, all the cadres included in the study were available in the health centres/polyclinics and primary hospital/district hospitals. Hence, it was deemed appropriate to compare the activity standards between health centres/polyclinics and primary hospitals/district hospital using an independent *t*-test (Table 6). The analysis showed that out of 67 health service activities examined across eight health professional groups, there was statistically significant differences in 12 of the activity standards between health centres/polyclinics and district/primary hospitals. This represents significant practice variation across 18.9% of health service activities. The practice variations were much more pronounced amongst activities performed by PAs (7 out of 10 activities). PAs spend significantly lower amount of time on patients at the district/primary hospitals compared to health centres for assessment, diagnosis and treatment of new cases (*MD* = −9, *p* < 0.001); review of follow-up patients (*MD* = −5, *p* = 0.002), minor surgical procedures (*MD* = −7, *p* = 0.003) as well as interventions for moderate-to-severe medical emergencies (*MD* = −8, *p* = 0.035). For General Practitioners, there was no statistically significant difference between the time spent on the various health service activities in health centres/polyclinic compared to the district/primary hospitals. Similarly, no statistically significant difference was seen for activities performed by midwives except for time spent on supervised vaginal delivery. In health centres/polyclinics, midwives spend an average of 147 min on women during vaginal delivery as compared to an average of 70 min spent in the district/primary hospital settings. The mean difference of 78 min between health centres/polyclinics and district hospitals/primary hospitals is statistically significant (*t* = −2.64, *p* = 0.013). With regard to clinical nurses, the time spent on consultation (where they were permitted to do) varied between district hospitals and health centres/polyclinics (*15 min vs. 12 min, p = 0.011*). For CHNs, only the time spent on growth monitoring significantly varied between district hospitals and health centres (*8 min vs. 5 min, p = 0.009*). In the case of Laboratory Scientist/Technicians, the *t*-test shows a significant difference between the time taken to conduct blood sugar test in the district/primary hospitals versus those of the health centres/polyclinics (*16 min vs. 9 min, p = 0.031*) as well as culture and sensitivity (*81 min vs. 64 min, p = 0.010*). Whereas no *t*-test was conducted in respect of activities performed by Nutritionist/Dietician due to sample size limitation, those of the Pharmacist/Pharmacy Technician was not statistically significant. 

### 3.5. Relationship between Health Service Activity Standards and Workload Levels and Availability of Tools/Equipment

Using a five-point Likert’s scale, health professionals were asked to self-rate the degree of workload in their health facility, workload level in their unit, and the availability of tools/equipment for work. The results show health facilities’ workload was 3.91 out of 5, which represents 78.2% (95% CI: 76.8–79.6%); and 4.01 out of 5 at the unit level (80.2%, 95% CI: 78.7–81.7%). Also, the availability of equipment was self-rated as 2.83 out of 5, which represents only 56.6% (95% CI: 54.8–58.4%) availability of the requisite tools and equipment. 

Using Pearson’s correlation, we explored the relationship between the main health service activity standards on the one hand and self-rated health facility workload level, unit workload level and availability of tools/equipment on the other hand (Table 7). The analysis showed that for General Practitioners, none of the variables (i.e., self-rated health facility workload level, unit workload level and availability of tools/equipment) had a statistically significant association with the activity standards. However, health facilities’ workload level had a moderate negative relationship with the time PAs spend on patient education/counselling (*r* = −0.314, *p* < 0.05). Similar moderate but statistically significant negative association are observed between workload at the unit level and PAs consultations for new outpatients (*r* = −0.303, *p* < 0.05); consultation for old outpatient cases (*r* = −0.319, *p* < 0.05) and review of inpatients (*r* = −0.351, *p* <0.05). Additionally, PAs time spent on minor surgical procedures significantly reduced with increasing workload level at the unit (*r* = −0.348, *p* < 0.05). Similarly, increasing availability of tools/equipment significantly reduced the time spent on minor surgical procedures (*r* = −0.355, *p* < 0.05) and referrals (*r* = −0.444, *p* < 0.001). On the other hand, the time PAs spent on interventions for critically ill medical emergencies rather increased with increasing levels of overall workload in the health facility (*r* = 0.414, *p* < 0.05).

For midwives, workload at the health facility moderately and positively correlated with the time spent on postnatal care consultations (*r* = 0.313, *p* < 0.05), family planning (*r* = 0.434, *p* < 0.05); inpatient care for the newborn (*r* = 0.354, *p* < 0.05); patient education/counselling (*r* = 0.454, *p* < 0.05) and management of complication of pregnancy (*r* = 0.422, *p* < 0.05). Availability of tools/equipment also had a moderate but statistically significant negative association with the time Clinical Nurses spend on the routine care of inpatients with moderate dependency (*r* = −0.333, *p* < 0.001). Noteworthy is also the Laboratory Scientists/Technicians spending more time on routine stool examination if there are tools/equipment and vice versa (*r* = 0.288, *p* < 0.05) but the time spent on full blood count being positively correlated with higher workload in the unit (*r* = 0.289, *p* < 0.05); same for blood sugar test (*r* = 0.327, *p* < 0.05) and blood chemistry analysis (*r* = 0.294, *p* < 0.05). Surprisingly, none of the health service activities performed by Pharmacists/Pharmacy Technicians had a statistically significant correlation with either self-rated workload levels or availability of the tools and equipment.

## 4. Discussion

To the best of our knowledge, this study is the first attempt to use a cross-sectional survey to estimate activity standards of health service activities across multiple health professions. Previous studies [5,9,10,34,35,36] have either been focused on only a few cadres and activities or based on expert group consensus to determine the standard time which the statistical uncertainty in the estimates could not be ascertained. The findings of the study demonstrate that using a cross-sectional survey could yield fairly reliable activity standards suitable for health workforce planning, just as the consensus-based estimates advocated in the WISN methodology. The added advantage of using a more rigorous survey approach which allows for sensitivity analysis in the planning process using the estimates of uncertainty, cannot be overemphasised. 

The study revealed that at the PHC settings, General Practitioners and Physician Assistants spent an average of 16 min, with the true estimate between 13 and 19 min for the assessment, diagnosis, and treatment of new patients in the outpatient clinic but 10 min (95% CI: 8–11 min) for patients on follow-up visits. The WISN study conducted in Ghana in 2015 [31] set a standard of 15 min for new outpatient cases at the primary hospitals and 8 min at the health centres/polyclinics for Physician Assistants, while that of the General Practitioners was 10 min in primary hospitals and 8 min in health centres/polyclinics. Comparing the result of the present study with the WISN results, there is no significant difference given that the WISN estimates fall within the confidence limits of the currents estimates. These do not only demonstrate the consistency of the results but also suggests that using a cross-sectional survey could (as in the case of the present study) be as reliable for health workforce planning as the consensus-based estimates advocated in the WISN methodology. Some studies have also found consultation time for doctors to be 18.21 min [37] which is also within the confidence limits of the present estimates. Anyhow, patients have indicated in other studies that a minimum consultation time of 6.3 min (or 9 min depending on the complexity) is necessary to keep them satisfied [38]. However, while the 2015 Ghana WISN results suggested an average of 105 min for major surgical procedures at the PHC levels, the current study estimates 64 min (95% CI: 44–84 min). The lack of convergence between the two studies in this instance is attributable to the wide diversity in the complexity of major surgeries. This is also reflected in the wide confidence interval in the current estimate, in which the confidence width is about 62.5% of the mean estimate. 

Although PAs spent about 6 min more on minor surgical procedures than General Practitioners, the difference between them is not statistically significant. When interpreted alongside the similarity in outpatient service time between these categories of health professionals, it appears that there may not be substantial variations in the quality of their services. Although previous studies [39,40] demonstrated that they do not necessarily differ in terms of the patient outcomes from their services, issues of subjective quality have not received much intellectual examination, and these findings may be one step towards that discourse. Furthermore, the analysis revealed a pronounced practice variation amongst PAs such that they spend more time on patients in health centres/polyclinics than district/primary hospitals. In health centres/polyclinics, PAs are at the helm of most clinical decision making where they need to spend more time for thorough assessment and procedures that they would otherwise easily refer (or pass on) to a physician in district/primary hospitals. The correlation analysis also revealed that improving the availability of tools/equipment significantly reduced the PAs time spent on minor surgical procedures by 35.5% (*p* < 0.05) and clinical nurses time spent on moderate dependency patients by 33.3% (*p* < 0.05). This finding points to the potential benefit of improving the availability of tools/equipment, which was rated to be only 57% by the health professionals. 

The present study found that midwives’ antenatal care consultation took an average of 22 min (95% CI: 18–26 min) is not significantly different from 25 min estimated from the previous WISN study in Ghana and 20–30 min estimated in a Namibian WISN study [35]. Additionally, whereas the current study shows that midwives spend 131 min (95% CI: 101–160 min) per supervised vaginal delivery, the WISN study in Ghana, which employed a time-motion observation for this activity, also estimated 152 min, which falls within the confidence limits of the present study. In the absence of well-conducted time-motion observations, the Namibian WISN study assumed 240 min per supervised vaginal delivery, but that is without considering the need to count only the direct patient care (or contact) time, not necessarily the entire length of the process of labour and delivery. In health centres/polyclinics, midwives spent an average of 78 min more on women during vaginal delivery as compared to district/primary hospital settings (*p* = 0.013). In the context of Ghana, there are very few women who deliver in health centres where they usually record one or two per day. As a result, midwives at health centres who usually reside within walking distance of the health centre seem to pay greater attention to the monitoring and psychosocial support to the pregnant in labour. 

We found that routine nursing care activities (made up of patient monitoring, administration of prescribed medications and support for patients’ activities of daily living) was an average of 40 min per inpatient day (95% CI: 38–42 min) for low acuity patients; 43 min (95% CI: 40–46 min) per inpatient day for moderate acuity patients and 135 min (95% CI: 127–144 min) per inpatient day for high dependency patients. These were, however, negatively correlated with the availability of tools/equipment, which suggests that clinical nurses would spend more time on direct routine care to inpatients if they had sufficient tools and equipment. Indeed, the resource constraints in the PHC setting, especially in health facilities and their effects on inpatient care, is well documented [17]. WISN studies in Kenya, Nigeria and Namibia [9,10,35] found lower estimates of time spent on routine nursing care, but the Ghana WISN study made similar or slightly higher estimates. The aforesaid further reinforces the point that health service activity standards cannot be universal but context-specific, taking into account models of care and mix of patient acuity levels [7]. 

Performing a full blood count analysis using an automated analyser machine is estimated to take 6 min (95% CI: 5–7 min), while it takes an average of 17 min (95% CI: 16–18 min) to conduct a rapid diagnostic test for malaria, HIV and similar ones routinely carried out in PHC settings. These estimates are not only consistent with the results of the Ghana WISN study but also similar to estimates from a Kenyan study [9], a similarity that may be a reflection of the highly standardised nature of laboratory processes and machines.

The correlation analysis revealed that a unit increase in perceived or actual workload was associated with a 31–44% reduction in the time PAs spend on the various health service activities; a similar finding was found for some activities undertaken by clinical nurses. These suggest that when workload increases, health professionals with the moral duty not to turn patients away rather reduces the time spent on patients to be able to attend to all the patients. This, however, have huge implications for quality of care and patient safety. 

The analysis shows that General Practitioners (who are not heads of health facilities) spend at least 3 hours per week or 7.5% of their working time on clinical meetings, while nurses and midwives spend up to 14% on both clinical meetings and handing over (or taking over) from one shift to another. When blood donation campaigns are taken into account, laboratory scientist spends up to 22.5% of their time on catalytic or support activities while Pharmacist spends at least 35% of their time on activities that are essential but not measurable per patient. These underscore the need to factor support activities into all health workforce planning models, especially needs-based planning tools that are regarded as the most conceptually appropriate and intuitively consistent planning framework for the tenets of UHC [41,42,43]. 

## 5. Limitations

Although the study used a cross-sectional study design with a carefully selected nationally representative sample, only six nutritionists/dieticians (out of an all-inclusive target of 23) responded to the survey, which requires the exercise of caution when interpreting or using the results of that category of health professionals. Linked to the sampling, the study was based on public health facilities and hence, excluded the private sector, some of which is known for low standards of care in Ghana [17,43]. Besides, the study adopted a self-reported approach which undoubtedly, could have benefited from a follow-up time-motion observation to validate the estimated time provided by health professionals. However, as time-motion studies are quite expensive, its use in this study was constrained by logistical challenges coupled with restrictions occasioned by the COVID−19 pandemic. 

## 6. Conclusions

The study systematically estimated the service standards (the mean estimates of time spent on health service activities), identifying statistically significant differences between health centres/polyclinics and district/primary hospitals in 18.9% (12 out of 67) of health service activities performed across eight health professional groups. For example, the standard workload for General Practitioners in PHC settings is 6030 new patients per year (or 10,444 follow-up cases per year); 6108 new outpatients per year for Physician Assistants, while midwives can conduct 735 spontaneous vaginal deliveries per year. In patients with low-to-moderate dependency, Clinical Nurses’ standard workload is between 2078 and 2549 per year, or a nurse-patient ratio of one is to 5–7 inpatients; and a CHN could give roughly 16,000 immunisations per year. 

The intensity of workload in health facilities was rated to be 3.91 out of 5 (78.2%) but as workload increases, and without a commensurate increase in staffing, health professionals reduced the time spent on individual patient care so as to be able to attend to all patients—a practice that could have adverse implications for quality of care and patient safety. Availability of tools and equipment at PHC was rated 2.83 out of 5 (56.6%) which a unit improvement is associated with a significant reduction in the time PAs spend on minor surgical procedures by 35.5% (*p* < 0.05) and clinical nurses time spent on moderate dependency patients by 33.3% (*p* < 0.05). 

## 7. Implications for Policies, Planning and Further Research

With the estimated standard workloads, it would be imperative to use them for evidence-based planning by estimating the optimal number of health professionals needed in Ghana’s PHC system and the consequent adjustments necessary in health professions educations to fill any gaps, and the budgetary requirements for their employment. The evidence also suggests that increasing workload levels were associated with reduced health professionals’ contact time with patients in many health service activities, which may be an indicator of the quality of healthcare provided. It will be important to further explore the potential relationship between workload and both objective and subjective measures of primary healthcare quality. 

The study partly demonstrates that improving the availability of tools and equipment could substantially improve staff efficiency, which ultimately lessens the need for additional staff. This provides some justification that can be used alongside other evidence for retooling the PHC system in Ghana. From a planning methodology perspective, the study revealed that health professionals spent between 7.5% and 35% of their time performing support activities that catalases the performance of direct patient care activities. These must be considered in all health workforce planning models, especially those that are founded on the needs-based framework—which has hitherto not taken support activities into account.

Based on the findings and the limitations identified, it is recommended that a similar study is executed in the private sector to establish homogeneity or otherwise in the service standards (and standard workloads) across the public and private sectors. Finally, a form of observation (preferably time-motion in nature) can be included as part of future data gathering process should the COVID-19 situation allow for direct observation, which was not feasible in this study due to COVID-19 restrictions and logistical challenges. 

## Figures and Tables

**Table 1:** Sample size and sample allocation.

Category of Staff	Greater Accra Region				Bono East Region				Upper East Region				Overall Sample
	PH	HC/PolyC	CHPS	Regional Sample	PH	HC/PolyC	CHPS	Regional Sample	PH	HC/PolyC	CHPS	Regional Sample	
Medical officers (general practitioners)	24	12	0	36	9	1	0	10	14	1	0	15	61
Professional nurses	30	29	1	60	7	3	10	20	13	12	4	29	109
Enrolled nurses	20	31	3	54	5	9	4	18	10	14	9	33	105
Community health nurses	9	26	18	53	10	6	8	24	1	11	20	32	109
Midwives	15	23	1	39	2	3	1	6	4	5	4	13	58
Pharmacists and pharmacy technicians	15	6	0	21	5	1	0	6	7	1	0	8	35
Dieticians and Nutritionists	10	11	0	21	1	0	0	1	0	1	0	1	23
Physician assistants	2	4	1	7	15	20	1	36	14	1	7	22	65
Laboratory scientists and technicians	20	5	0	25	4	2	0	6	16	0	0	16	47
Total	92	127	25	316	54	44	25	127	63	46	44	169	612

PH = Primary hospital; HC = Health centre; PolyC = Polyclinic; CHPS = Community-Based Health Planning and Services.

**Table 2:** Age and gender distribution of health professionals.

Age Bracket	Frequency	Percentage
20–29 Years	166	33.0%
30–39 Years	267	53.1%
40–49 years	49	9.7%
50–59 Years	20	4.0%
60 Years or more	1	0.2%
Total	503	100.0%
Gender		
Male	185	36.8%
Female	318	63.2%
Total	503	100.0%

**Table 3:** The professional background of participants.

Variable	Dimension					Frequency	Percent
Type of Health Professional	General Practitioner (Generalist Doctor)					30	6.0%
	Physician Assistant					47	9.3%
	Midwife					47	9.3%
	Clinical Nurse (General Nurse/Enrolled Nurse)					219	43.5%
	Preventive Nurse (Community Health Nurse)					65	12.9%
	Nutritionist/ Dietician					6	1.2%
	Laboratory Scientist/Technician					56	11.1%
	Pharmacist/Pharmacy Technician					33	6.6%
Total						503	100.0%
Highest qualification of the Health Professional	Certificate					158	31.4%
	Diploma					133	26.4%
	Post-Basic (Advanced) Diploma					30	6.0%
	First Degree					132	26.2%
	Masters/post-graduate specialisation					26	5.2%
	Other					24	4.8%
Total						503	100.0%
**Variable**	**Min.**	**Max.**	**Mean (Years)**	**Std. Error**	**SD**	**95% CI**	
						**Lower**	**Upper**
Years of experience in the profession	1.0	44.0	6.8	0.29	6.57	6.3	7.4
Years of experience in the current health facility	1.0	23.0	3.7	0.15	3.41	3.4	4.0

**Table 4:** Health service activity standards estimated by health professionals in primary health care settings.

Category of Health Professional	Descriptive Statistics	Unit of Measurement	Service Standard Time (Mean)	Std. Error of the Mean	Std. Deviation	95% Confidence Interval	
						Lower	Upper
General Practitioner (Generalist Doctor)	Assessment, diagnosis and treatment of a new out-patient case	Minutes per patient	16	1.63	8.75	13	19
	Review of a follow-up out-patient case (old cases)	Minutes per patient per visit	9	0.93	5.00	7	11
	Review of inpatient per patient day (daily ward rounds)	Minutes per patient per inpatient day	15	1.55	8.19	12	18
	Referral of a patient	Minutes per patient	14	1.84	9.58	10	17
	Minor surgical procedures (e.g., suturing lacerations, incision and drainage)	Minutes per case	19	1.74	9.02	16	23
	Major surgical procedures	Minutes per case	64	10.13	49.61	44	84
	Patient education and counselling	Minutes per patient	14	1.54	8.29	11	17
	Interventions for minor (simple) medical emergencies	Minutes per case	12	1.37	7.23	10	15
	Interventions for moderate-to-severe medical emergencies	Minutes per case	25	3.17	17.09	19	31
	Interventions for critically ill medical emergencies	Minutes per case	35	3.74	19.81	28	42
	Clinical meetings	Hours per week	3	0.31	1.59	2	3
Physician Assistant (Medical)	Assessment, diagnosis and treatment of a new out-patient case	Minutes per patient	16	1.28	8.59	13	18
	Review of a follow-up out-patient case (old cases)	Minutes per patient per visit	10	0.83	5.59	8	11
	Review of inpatient per patient day (daily ward rounds)	Minutes per patient per inpatient day	14	1.27	7.39	12	16
	Referral of a patient	Minutes per patient	13	1.15	7.72	10	15
	Minor surgical procedures (e.g., suturing lacerations, incision and drainage)	Minutes per case	25	1.48	9.94	22	28
	Patient education and counselling	Minutes per patient	11	0.99	6.64	9	12
	Interventions for minor (simple) medical emergencies	Minutes per case	17	1.68	11.24	14	20
	Interventions for moderate-to-severe medical emergencies	Minutes per case	25	2.71	18.18	20	31
	Interventions for critically ill medical emergencies	Minutes per case	27	2.76	17.02	21	32
	Clinical meetings	Hours per week	3	0.28	1.75	2	3
Midwife	Antenatal care (ANC) consultation	Minutes per patient per visit	22	2.03	13.63	18	26
	Postnatal care (PNC) consultation	Minutes per patient per visit	19	1.24	8.20	17	22
	Family planning service (non-invasive procedure)	Minutes per patient per visit	16	2.31	12.67	12	21
	Family planning service (invasive procedure)	Minutes per patient per visit	39	2.95	13.21	33	45
	Prevention of Mother-To-Child (PMTC) transmission of HIV during antenatal care visit	Minutes per patient per visit	17	1.30	8.52	14	19
	Vaginal delivery	Minutes per patient	131	15.18	97.23	101	160
	Inpatient care per patient day (routine care for mother)	Minutes per patient per inpatient day	30	2.83	16.51	24	36
	Inpatient care per patient day (routine care for new-born)	Minutes per patient per inpatient day	30	3.29	18.62	24	36
	Admission processes per patient	Minutes per patient	22	1.45	8.34	19	24
	Discharge processes per patient	Minutes per patient	21	1.54	8.83	18	24
	Preparing a patient for caesarean section	Minutes per case	32	3.23	14.06	26	38
	Patient education and counselling	Minutes per patient	23	1.00	6.45	21	25
	In-patient management of complications of pregnancy	Minutes per patient per inpatient day	44	2.24	12.86	40	49
	Daily report writing	Minutes per day	40	3.47	21.42	33	46
	Monthly reports	Hours per month	7	0.99	6.57	5	9
	Taking-over and handing-over	Minutes per day	31	2.94	17.42	25	36
	Clinical meetings	Hours per week	3	0.36	2.10	2	4
Clinical Nurse (Registered General Nurse & Enrolled Nurse)	Out-patient care (triaging, vital signs and history taking)	Minutes per patient per visit	10	0.26	3.85	9	10
	Out-patient consultation (where applicable)	Minutes per patient per visit	13	0.53	5.24	12	14
	Admission processes per patient	Minutes per patient	19	0.55	6.98	18	20
	Discharge processes per patient	Minutes per patient	16	0.75	9.37	14	17
	Pre-Operative preparation of patients	Minutes per case	26	1.50	16.65	23	28
	Post-operative management which is different from routine care	Minutes per case	42	2.11	23.35	38	46
	Inpatient care per patient day (routine care) for low dependent cases or mildly ill patients	Minutes per patient per inpatient day	40	1.06	14.82	38	42
	Inpatient care per patient day (routine care) for moderately dependent cases or patients with severe illness	Minutes per patient per inpatient day	43	1.54	21.29	40	46
	Inpatient care per patient day (routine care) for highly dependent cases or critically ill patients	Minutes per patient per inpatient day	135	4.27	55.64	127	144
	Discharge patient education and counselling	Minutes per patient	18	0.56	8.07	17	19
	Minor surgical procedures (suturing lacerations, incision and drainage, wound dressings)	Minutes per patient	27	1.20	16.74	24	29
	Daily report writing	Minutes per day	32	1.98	25.32	29	36
	Monthly reports	Hours per month	5	0.31	3.63	4	6
	Taking-over and handing-over	Minutes per day	25	1.15	16.20	23	28
	Clinical meetings	Hours per week	3	0.13	1.64	3	3
Preventive Nurse (Community Health Nurse)	Family planning	Minutes per patient per visit	14	1.18	8.93	12	17
	Out-patient consultation to manage minor ailments	Minutes per patient per visit	12	1.16	6.54	10	14
	Referral of patients	Minutes per patient	9	0.92	6.40	8	11
	Patient education and counselling	Minutes per patient	14	1.37	10.82	11	17
	Home visiting	Minutes per home visit	23	1.49	11.92	20	26
	School health	Minutes per patient	12	1.01	7.88	10	14
	Immunisation	Minutes per patient	6	0.44	3.56	5	7
	Growth monitoring	Minutes per patient	6	0.37	2.95	6	7
	Monthly reports	Hours per month	4	0.28	2.15	4	5
	Cold chain management	Minutes per day	17	1.47	11.20	14	19
	Clinical meetings	Hours per week	2	0.10	0.66	2	2
Nutritionist and Dietician	Nutritional Status Assessment	Minutes per patient	13	4.60	11.26	4	22
	Patient education and counselling	Minutes per patient	22	2.79	6.83	16	27
	Referral of patients	Minutes per patient	8	2.18	4.88	3	12
	Intervention including diet planning for patients	Minutes per patient per visit	27	7.84	17.54	12	42
	School health	Minutes per patient	20	5.77	10.00	9	31
	Follow-ups/home visits	Minutes per patient	23	10.93	18.93	2	45
	Monthly reports	Hours per month	47	28.01	68.62	−8	102
	Clinical meetings	Hours per week	3	0.88	1.53	1	4
Laboratory Scientist and Laboratory Technician	Sample taking and processing	Minutes per sample	9	0.67	4.75	8	11
	Full blood count (using an automated machine)	Minutes per sample	6	0.41	3.06	5	7
	Rapid diagnostic tests (Malaria, HIV, etc.)	Minutes per sample	17	0.53	3.89	16	18
	Malaria test using microscopy	Minutes per sample	27	1.53	11.15	24	30
	Urine routine examination	Minutes per sample	18	0.99	7.30	16	20
	Stool Urine routine examination	Minutes per sample	23	1.83	13.41	20	27
	Blood sugar test	Minutes per sample	14	1.39	10.30	11	16
	Blood donor bleeding	Minutes per patient	31	1.24	8.59	29	34
	Preparing blood for transfusion	Minutes per unit of blood	35	1.34	9.06	32	37
	Blood chemistry	Minutes per batch of samples	17	0.65	4.33	16	18
	Culture and sensitivity analysis	Minutes per sample	74	3.43	15.36	67	81
	Report writing	Minutes per day	11	0.74	4.98	10	13
	Blood donation campaign	Hours per month	6	0.84	4.26	5	8
	Clinical meetings	Hours per week	3	0.77	4.70	2	5
Pharmacist & Pharmacy Technician	Prescription auditing and dispensing for out-patient cases	Minutes per patient per visit	7	0.71	4.05	6	9
	Prescription auditing and dispensing for in-patient cases	Minutes per patient per inpatient day	7	0.52	2.96	6	8
	Prescription refilling for chronic conditions	Minutes per patient per visit	7	1.04	5.50	5	9
	Pharmaceutical interventions to correct prescription errors	Minutes per case	7	0.73	4.12	6	9
	Patient adherence counselling and education	Minutes per patient	13	0.90	5.19	11	15
	Report writing	Minutes per day	24	1.32	6.20	21	26
	Reconstitution of powdered preparations	Hours per week	3	0.40	1.77	2	4
	Clinical meetings	Hours per week	4	1.24	5.95	1	6
	Management of stocks	Hours per week	7	1.05	5.74	5	9
	Procurement activities	Hours per quarter	16	3.69	15.64	8	23
	Quality Assurance (QA), Drug and Therapeutics Committee (DTC) activities	Hours per quarter	4	1.04	3.90	2	6

**Table 5:** Estimated standard workload per year for health service activities.

Category of Health Professional	Descriptive Statistics	Standard Workload per Year	95% Confidence Interval	
			Best Case Scenario (Using Lower Bound of the Service Standard)	Worst Case Scenario (Using the Upper Bound of the Service Standard)
General Practitioner (Generalist Doctor)	Assessment, diagnosis and treatment of a new out-patient case	6030	7558	5017
	Review of a follow-up out-patient case (old cases)	10,444	13,053	8704
	Review of inpatient per patient day (daily ward rounds)	6319	7915	5259
	Referral of a patient	6973	9489	5511
	Minor surgical procedures (e.g., suturing lacerations, incision and drainage)	4953	6020	4207
	Major surgical procedures	1488	2159	1135
	Patient education and counselling	6823	8708	5608
	Interventions for minor (simple) medical emergencies	7689	9817	6319
	Interventions for moderate-to-severe medical emergencies	3838	5126	3068
	Interventions for critically ill medical emergencies	2721	3444	2249
Physician Assistant (Medical)	Assessment, diagnosis and treatment of a new out-patient case	6108	7283	5259
	Review of a follow-up out-patient case (old cases)	9921	11,962	8475
	Review of inpatient per patient day (daily ward rounds)	6789	8254	5765
	Referral of a patient	7567	9224	6415
	Minor surgical procedures (e.g., suturing lacerations, incision and drainage)	3802	4301	3406
	Patient education and counselling	9043	11,088	7635
	Interventions for minor (simple) medical emergencies	5627	6985	4711
	Interventions for moderate-to-severe medical emergencies	3739	4726	3093
	Interventions for critically ill medical emergencies	3541	4435	2947
Midwife	Antenatal care (ANC) consultation	4449	5455	3755
	Postnatal care (PNC) consultation	5010	5735	4448
	Family planning service (non-invasive procedure)	5952	8280	4645
	Family planning service (invasive procedure)	2446	2869	2132
	Prevention of Mother-To-Child (PMTC) transmission of HIV during antenatal care visit	5694	6708	4946
	Vaginal delivery	735	951	598
	Inpatient care per patient day (routine care for mother)	3200	3926	2700
	Inpatient care per patient day (routine care for new-born)	3200	4077	2634
	Admission processes per patient	4436	5108	3921
	Discharge processes per patient	4533	5284	3968
	Preparing a patient for caesarean section	3015	3762	2515
	Patient education and counselling	6892	8796	5665
	In-patient management of complications of pregnancy	2163	2400	1968
Clinical Nurse (Registered General Nurse & Enrolled Nurse)	Out-patient care (triaging, vital signs and history taking)	9786	10,326	9299
	Out-patient consultation (where applicable)	7373	8009	6831
	Admission processes per patient	4436	5108	3921
	Discharge processes per patient	4533	5284	3968
	Pre-Operative preparation of patients	3763	4254	3374
	Post-operative management which is different from routine care	2285	2535	2080
	Inpatient care per patient day (routine care) for low dependent cases or mildly ill patients	2416	2549	2296
	Inpatient care per patient day (routine care) for moderately dependent cases or patients with severe illness	2224	2391	2078
	Inpatient care per patient day (routine care) for highly dependent cases or critically ill patients	709	756	668
	Discharge patient education and counselling	5324	5672	5017
	Minor surgical procedures (suturing lacerations, incision and drainage, wound dressings)	3586	3932	3296
Preventive Nurse (Community Health Nurse)	Family planning	6713	8012	5777
	Out-patient consultation to manage minor ailments	7895	9703	6655
	Referral of patients	10,289	12,768	8617
	Patient education and counselling	6892	8796	5665
	Home visiting	4237	4863	3753
	School health (assessment of pupil)	7799	9291	6719
	Immunisation	16,000	18,714	13,973
	Growth monitoring	15,335	17,320	13,759
Nutritionist and Dietician	Nutritional Status Assessment	7202	22,203	4298
	Patient education and counselling	6892	8796	5665
	Referral of patients	10,289	12,768	8617
	Intervention including diet planning for patients	3556	8255	2266
	School health	4800	11,056	3065
	Follow-ups/home visits	4115	50,284	2145
Laboratory Scientist and Laboratory Technician	Sample taking and processing	10,116	11,726	8894
	Full blood count (using an automated machine)	16,667	19,392	14,613
	Rapid diagnostic tests (Malaria, HIV, etc.)	5511	5863	5199
	Malaria test using microscopy)	3539	3979	3186
	Urine routine examination	5206	5820	4709
	Stool routine examination	4124	4872	3574
	Blood sugar test	7012	8753	5849
	Blood donor bleeding	3057	3314	2838
	Preparing blood for transfusion	2777	3004	2582
	Blood chemistry	5707	6171	5309
	Culture and sensitivity analysis	1297	1427	1189
Pharmacist & Pharmacy Technician	Prescription auditing and dispensing for out-patient cases	13,205	16,304	11,096
	Prescription auditing and dispensing for in-patient cases	13,953	16,396	12,144
	Prescription refilling for chronic conditions	13,994	19,910	10,788
	Pharmaceutical interventions to correct prescription errors	13,187	16,407	11,023
	Patient adherence counselling and education	7541	8761	6620

Note: The calculation for standard workload assumes that the health professional dedicates all his/her available working time to only a particular activity throughout the year.

**Table 6:** Test of differences in activity standards between health centres/polyclinics and primary/district hospitals.

Health Professional	Health Service Activities	Mean Time (Activity Standard)			*t*-Test for Equality of Means					
		Unit of Measurement	Primary/District Hospital (a)	Health Centre/Polyclinic (b)	Mean Difference (a-b)	Std. Error Difference	95% Confidence Interval of the Difference		*t*-Statistic	*p*-Value (2-Tailed)
							Lower	Upper		
General Practitioner (Generalist Doctor)	Assessment, diagnosis and treatment of a new out-patient case	Minutes per patient	16	10	6	5.29	−4	17	1.21	0.235
	Review of a follow-up out-patient case (old cases)	Minutes per patient per visit	9	8	1	3.10	−5	7	0.28	0.784
	Review of inpatient per patient day (daily ward rounds)	Minutes per patient per inpatient day	15	15	0	6.12	−13	13	0.01	0.995
	Referral of a patient	Minutes per patient	13	18	−4	7.13	−19	11	−0.59	0.563
	Minor surgical procedures (e.g., suturing lacerations, incision and drainage)	Minutes per case	20	13	7	6.61	−6	21	1.09	0.285
	Major surgical procedures	Minutes per case	67	33	34	36.75	−42	110	0.93	0.362
	Patient education and counselling	Minutes per patient	14	12	3	5.12	−8	13	0.49	0.626
	Interventions for minor (simple) medical emergencies	Minutes per case	13	10	3	5.39	−9	14	0.47	0.641
	Interventions for moderate-to-severe medical emergencies	Minutes per case	27	8	18	10.01	−2	39	1.83	0.078
	Interventions for critically ill medical emergencies	Minutes per case	37	13	24	14.03	−5	53	1.72	0.097
Physician Assistant (Medical)	Assessment, diagnosis and treatment of a new out-patient case	Minutes per patient	11	19	−9	2.25	−13	−4	−3.85	0.000
	Review of a follow-up out-patient case (old cases)	Minutes per patient per visit	7	12	−5	1.52	−8	−2	−3.30	0.002
	Review of inpatient per patient day (daily ward rounds)	Minutes per patient per inpatient day	10	17	−7	2.28	−12	−3	−3.20	0.003
	Referral of a patient	Minutes per patient	11	14	−4	2.28	−8	1	−1.63	0.111
	Minor surgical procedures (e.g., suturing lacerations, incision and drainage)	Minutes per case	21	28	−7	2.84	−12	−1	−2.38	0.022
	Patient education and counselling	Minutes per patient	8	12	−4	1.92	−8	0	−2.02	0.049
	Interventions for minor (simple) medical emergencies	Minutes per case	12	21	−8	3.17	−15	−2	−2.61	0.013
	Interventions for moderate-to-severe medical emergencies	Minutes per case	19	30	−11	5.24	−22	−1	−2.17	0.035
	Interventions for critically ill medical emergencies	Minutes per case	26	28	−2	5.59	−13	9	−0.38	0.709
Midwife	Antenatal care (ANC) consultation	Minutes per patient per visit	26	21	5	5.09	−5	15	0.95	0.347
	Postnatal care (PNC) consultation	Minutes per patient per visit	21	19	2	3.09	−4	8	0.59	0.556
	Family planning service (non-invasive procedure)	Minutes per patient per visit	23	15	8	6.04	−4	21	1.40	0.178
	Family planning service (invasive procedure)	Minutes per patient per visit	42	37	5	5.97	−8	17	0.75	0.461
	Prevention of Mother-To-Child (PMTC) transmission of HIV during antenatal care visit	Minutes per patient per visit	20	16	4	3.02	−2	10	1.29	0.205
	Vaginal delivery	Minutes per patient	70	147	−78	29.35	−137	−18	−2.64	0.013
	Inpatient care per patient day (routine care for mother)	Minutes per patient per inpatient day	28	31	−3	5.82	−15	9	−0.52	0.605
	Inpatient care per patient day (routine care for new-born)	Minutes per patient per inpatient day	34	27	8	6.60	−6	21	1.15	0.257
	Admission processes per patient	Minutes per patient	21	22	0	2.99	−6	6	−0.12	0.905
	Discharge processes per patient	Minutes per patient	22	20	2	3.15	−5	8	0.53	0.599
	Preparing a patient for caesarean section	Minutes per case	30	37	−7	7.35	−22	8	−0.95	0.354
	Patient education and counselling	Minutes per patient	24	23	1	2.43	−4	6	0.32	0.753
	In-patient management of complications of pregnancy	Minutes per patient per inpatient day	45	47	−3	5.09	−13	8	−0.49	0.627
Clinical Nurse (Registered General Nurse & Enrolled Nurse)	Out-patient care (triaging, vital signs and history taking)	Minutes per patient per visit	10	9	1	0.58	0	2	1.35	0.177
	Out-patient consultation (where applicable)	Minutes per patient per visit	15	12	3	1.29	1	6	2.61	0.011
	Admission processes per patient	Minutes per patient	19	19	0	1.29	−3	2	−0.24	0.812
	Discharge processes per patient	Minutes per patient	15	18	−3	1.75	−7	0	−1.75	0.082
	Pre-Operative preparation of patients	Minutes per case	25	28	−2	3.86	−10	5	−0.64	0.523
	Post-operative management which is different from routine care	Minutes per case	41	49	−8	5.47	−19	3	−1.49	0.140
	Inpatient care per patient day (routine care) for low dependent cases or mildly ill patients	Minutes per patient per inpatient day	40	40	−1	2.19	−5	4	−0.28	0.782
	Inpatient care per patient day (routine care) for moderately dependent cases or patients with severe illness	Minutes per patient per inpatient day	43	43	1	3.19	−6	7	0.17	0.866
	Inpatient care per patient day (routine care) for highly dependent cases or critically ill patients	Minutes per patient per inpatient day	141	123	18	9.23	0	36	1.96	0.051
	Discharge patient education and counselling	Minutes per patient	19	17	2	1.20	−1	4	1.25	0.212
	Minor surgical procedures (suturing lacerations, incision and drainage, wound dressings)	Minutes per patient	29	25	4	2.53	−1	9	1.64	0.104
Community Health Nurse	Family planning	Minutes per patient per visit	13	15	−1	3.30	−8	5	−0.41	0.686
	Out-patient consultation to manage minor ailments	Minutes per patient per visit	14	12	2	3.86	−6	11	0.58	0.571
	Referral of patients	Minutes per patient	8	9	−1	2.78	−7	5	−0.33	0.745
	Patient education and counselling	Minutes per patient	13	17	−4	4.42	−13	5	−0.96	0.345
	Home visiting	Minutes per home visit	25	21	4	4.01	−4	12	0.97	0.337
	School health	Minutes per patient	14	13	1	2.78	−5	6	0.28	0.784
	Immunisation	Minutes per patient	7	6	1	1.16	−1	3	0.90	0.375
	Growth monitoring	Minutes per patient	8	5	3	0.93	1	4	2.74	0.009
Laboratory Scientist/Technician	Sample taking and processing	Minutes per sample	10	9	1	1.47	−2	4	0.41	0.685
	Full blood count (using an automated machine)	Minutes per sample	6	5	0	0.90	−1	2	0.47	0.639
	Rapid diagnostic tests (Malaria, HIV, etc.)	Minutes per sample	17	18	−1	1.16	−4	1	−1.27	0.211
	Malaria test using microscopy)	Minutes per sample	29	24	5	3.30	−2	11	1.47	0.148
	Urine routine examination	Minutes per sample	18	20	−2	2.14	−6	2	−0.86	0.394
	Stool Urine routine examination	Minutes per sample	24	21	3	3.94	−4	11	0.89	0.379
	Blood sugar test	Minutes per sample	16	9	7	2.95	1	12	2.21	0.031
	Blood donor bleeding	Minutes per patient	32	29	3	2.77	−2	9	1.26	0.213
	Preparing blood for transfusion	Minutes per unit of blood	35	35	0	3.17	−6	6	0.01	0.993
	Blood chemistry	Minutes per batch of samples	17	18	−1	1.62	−4	2	−0.65	0.519
	Culture and sensitivity analysis	Minutes per sample	81	64	17	5.97	5	30	2.86	0.010
Pharmacist/Pharmacy Technician	Prescription auditing and dispensing for out-patient cases	Minutes per patient per visit	8	6	2	1.44	−1	5	1.11	0.276
	Prescription auditing and dispensing for in-patient cases Inpatient	Minutes per patient per inpatient day	7	7	0	1.10	−2	3	0.30	0.763
	Prescription refilling for chronic conditions	Minutes per patient per visit	8	5	4	2.10	−1	8	1.67	0.107
	Pharmaceutical interventions to correct prescription errors	Minutes per case	7	8	−1	1.52	−4	2	−0.67	0.508
	Patient adherence counselling and education	Minutes per patient	14	11	2	1.84	−2	6	1.21	0.237

**Table 7:** Factors influencing the average time taken to accomplish health service activities.

Health Professional	Health Service Activities	Facility Workload Level	Unit Workload Level	Availability of Tools and Equipment
General Practitioner (Generalist Doctor)	Assessment, diagnosis and treatment of a new out-patient case	0.006	0.04	0.07
	Review of a follow-up out-patient case (old cases)	−0.192	−0.14	−0.15
	Review of inpatient per patient day (daily ward rounds)	0.043	0.33	−0.01
	Referral of a patient	0.068	0.14	−0.21
	Minor surgical procedures (e.g., suturing lacerations, incision and drainage)	0.349	0.33	−0.16
	Major surgical procedures	0.171	0.33	−0.12
	Patient education and counselling	0.095	0.14	0.10
	Interventions for minor (simple) medical emergencies	−0.069	−0.02	−0.03
	Interventions for moderate-to-severe medical emergencies	−0.065	−0.02	0.13
	Interventions for critically ill medical emergencies	0.165	0.24	0.18
Physician Assistant (Medical)	Assessment, diagnosis and treatment of a new out-patient case	−0.099	−0.303 *	−0.25
	Review of a follow-up out-patient case (old cases)	−0.129	−0.319 *	−0.17
	Review of inpatient per patient day (daily ward rounds)	−0.042	−0.351 *	−0.339 *
	Referral of a patient	−0.044	−0.14	−0.444 **
	Minor surgical procedures (e.g., suturing lacerations, incision and drainage)	−0.283	−0.0348 *	−0.355 *
	Patient education and counselling	−0.314*	−0.04	0.27
	Interventions for minor (simple) medical emergencies	0.072	−0.07	−0.13
	Interventions for moderate-to-severe medical emergencies	0.107	−0.04	−0.07
	Interventions for critically ill medical emergencies	0.414 **	0.25	−0.01
Midwife	Antenatal care (ANC) consultation	0.254	0.11	0.22
	Postnatal care (PNC) consultation	0.313 *	0.21	0.10
	Family planning service (non-invasive procedure)	0.434 *	0.29	−0.12
	Family planning service (invasive procedure)	0.549 *	0.37	−0.08
	Prevention of Mother-To-Child (PMTC) transmission of HIV during antenatal care visit	0.192	−0.16	−0.15
	Vaginal delivery	−0.043	−0.19	−0.15
	Inpatient care per patient day (routine care for mother)	0.012	−0.21	−0.20
	Inpatient care per patient day (routine care for new-born)	0.354 *	0.07	−0.20
	Admission processes per patient	0.267	0.23	0.09
	Discharge processes per patient	0.319	0.33	0.02
	Preparing a patient for caesarean section	0.495 *	0.23	−0.09
	Patient education and counselling	0.454 **	0.06	0.14
	In-patient management of complications of pregnancy	0.422 *	0.22	0.26
Clinical Nurse (Registered General Nurse & Enrolled Nurse)	Out-patient care (triaging, vital signs and history taking)	0.056	−0.04	0.12
	Out-patient consultation	0.051	0.00	0.07
	Admission processes per patient	0.156 *	0.167*	0.00
	Discharge processes per patient	0.013	−0.02	0.05
	Pre-Operative preparation of patients	0.240 **	0.03	0.04
	Post-operative management which is different from routine care	0.169	0.268 **	−0.04
	Inpatient care per patient day (routine care) for low dependent cases or mildly ill patients	0.062	0.04	−0.02
	Inpatient care per patient day (routine care) for moderately dependent cases	−0.134	−0.03	−0.333 **
	Inpatient care per patient day (routine care) for highly dependent cases or critically ill patients	0.028	0.04	0.152 *
	Discharge patient education and counselling	−0.004	−0.01	−0.02
	Minor surgical procedures (suturing lacerations, incision and drainage, wound dressings)	0.181 *	0.10	0.03
Community Health Nurse	Family planning	0.227	0.24	0.16
	Out-patient consultation to manage minor ailments	−0.079	0.02	−0.14
	Referral of patients	−0.248	−0.26	0.09
	Patient education and counselling	0.157	0.19	−0.01
	Home visiting (minutes per each home visit)	−0.162	0.05	−0.03
	School health (minutes per each pupil)	0.175	0.04	0.18
	Immunisation (minutes per child immunised)	0.073	0.24	0.19
	Growth monitoring (minutes per child)	−0.045	0.21	0.17
Laboratory Scientist/Technician	Sample taking and processing	−0.189	−0.03	0.26
	Full blood count (using an automated machine)	0.214	0.289 *	0.06
	Rapid diagnostic tests (Malaria, HIV, etc.)	−0.128	−0.04	−0.15
	Malaria test using microscopy	−0.014	0.02	0.12
	Urine routine examination	0.032	0.07	−0.15
	Stool routine examination	0.116	0.24	0.288 *
	Blood sugar test	0.308 *	0.327 *	0.23
	Blood donor bleeding	−0.017	−0.05	−0.01
	Preparing blood for transfusion	0.036	0.05	−0.21
	Blood chemistry (minutes per sample)	0.304 *	0.294 *	0.28
	Culture and sensitivity analysis (minutes per sample)	0.093	0.16	0.22
Pharmacist/Pharmacy Technician	Prescription auditing and dispensing for out-patient cases	−0.064	0.06	0.16
	Prescription auditing and dispensing for in-patient cases	−0.272	−0.25	0.05
	Prescription refilling for chronic conditions	0.072	0.11	−0.06
	Pharmaceutical interventions to correct prescription errors	−0.064	0.28	0.10
	Patient adherence counselling and education	−0.301	−0.15	0.02

** Correlation is significant at the 0.01 level (2-tailed). * Correlation is significant at the 0.05 level (2-tailed). Facility workload level was measured on a scale of 0 to 5 (where 0 is no workload and 5 is the heaviest workload possible in any health facility); Unit workload level was measured on a scale of 0 to 5 (where 0 is no workload and 5 is the greatest workload possible in any Unit in the facility); Availability to tools and equipment was measured on a scale of 0 to 5 (where 0 is a situation all the tools/equipment are not available, and 5 is a situation that all tools/equipment are available

## Data Availability

The raw data is available with the authors and could be provided upon reasonable request, subject to the conditions of the ethics approval.

## Supplementary Materials

Supplementary Materials are available online at https://www.mdpi.com/2227-9032/9/3/332/s1.
