# Supplementary material for: Health Service Activity Standards and Standard Workloads for Primary Healthcare in Ghana: A Cross-Sectional Survey of Health Professionals
Source: Healthcare (Basel). 2021 Mar 16;9(3):332. doi: 10.3390/healthcare9030332 (PMC8000167; doi:10.3390/healthcare9030332)
Supplement: Supplementary file 1 [file healthcare-09-00332-s001.pdf]

Supplementary table: Available Working Time of the Health Professionals.

Category of Health Professional	Public Holidays	Annual Leave	Average Sick and other leave days	Average Training Days per Year	Weekend days	Total Non-working Days	Available Working Days	Available Working Time in Hours	Available Working Time in Minutes
General Practitioner (Generalist Doctor)	13	36	7	7	104	167	198	1,584	95,040
Physician Assistant (Medical)	13	36	7	7	104	167	198	1,584	95,040
Midwife	13	36	7	5	104	165	200	1,600	96,000
Clinical Nurse (Registered General Nurse & Enrolled Nurse)	13	36	7	5	104	165	200	1,600	96,000
Preventive Nurse (Community Health Nurse)	13	36	7	5	104	165	200	1,600	96,000
Nutritionist and Dietician	13	36	7	5	104	165	200	1,600	96,000
Laboratory Scientist and Laboratory Technician	13	36	7	5	104	165	200	1,600	96,000
Pharmacist & Pharmacy Technician	13	36	7	5	104	165	200	1,600	96,000

Data sources: Ghana Health Service Annual reports and Ghana WISN study report (2015).
